# Space-Time Clustering of Non-Hodgkin Lymphoma Using Residential Histories in a Danish Case-Control Study

**DOI:** 10.1371/journal.pone.0060800

**Published:** 2013-04-01

**Authors:** Rikke Baastrup Nordsborg, Jaymie R. Meliker, Annette Kjær Ersbøll, Geoffrey M. Jacquez, Ole Raaschou-Nielsen

**Affiliations:** 1 Danish Cancer Society Research Center, Copenhagen, Denmark; 2 National Institute of Public Health, University of Southern Denmark, Copenhagen, Denmark; 3 Department of Preventive Medicine and Graduate Program in Public Health, Stony Brook University, Stony Brook, New York, United States of America; 4 BioMedware Inc., Ann Arbor, Michigan, United States of America; 5 State University of New York at Buffalo, Buffalo, New York, United States of America; Karolinska Institutet, Sweden

## Abstract

Non-Hodgkin lymphoma (NHL) is a frequent cancer and incidence rates have increased markedly during the second half of the 20^th^ century; however, the few established risk factors cannot explain this rise and still little is known about the aetiology of NHL. Spatial analyses have been applied in an attempt to identify environmental risk factors, but most studies do not take human mobility into account. The aim of this study was to identify clustering of NHL in space and time in Denmark, using 33 years of residential addresses. We utilised the nation-wide Danish registers and unique personal identification number that all Danish citizens have to conduct a register-based case-control study of 3210 NHL cases and two independent control groups of 3210 each. Cases were identified in the Danish Cancer Registry and controls were matched by age and sex and randomly selected from the Civil Registration System. Residential addresses of cases and controls from 1971 to 2003 were collected from the Civil Registration System and geocoded. Data on pervious hospital diagnoses and operations were obtained from the National Patient Register. We applied the methods of the newly developed Q-statistics to identify space-time clustering of NHL. All analyses were conducted with each of the two control groups, and we adjusted for previous history of autoimmune disease, HIV/AIDS or organ transplantation. **S**ome areas with statistically significant clustering were identified; however, results were not consistent across the two control groups; thus we interpret the results as chance findings. We found no evidence for clustering of NHL in space and time using 33 years of residential histories, suggesting that if the rise in incidence of NHL is a result of risk factors that vary across space and time, the spatio-temporal variation of such factors in Denmark is too small to be detected with the applied method.

## Introduction

Non-Hodgkin lymphoma (NHL) is the 11^th^ most frequent cancer among men and women worldwide [1] and accounts for three percent of all new cancer cases [2]. Incidence rates have increased markedly during the second half of the 20^th^ century [3], although rates have stabilized since the mid-1990’s [4]. The same trend is seen in Denmark, where the age standardized incidence rate (world standard population) was 5.5 per 100,000 person-years for men and 3.6 for women in 1975. In 1995 the rates had doubled to 11.1 for men and 7.5 for women, respectively [5]. High incidence rates of NHL are found in North America, Europe, Australia and New Zealand and some parts of Africa, whereas low rates are observed in Eastern and Southern Asia [1].

Despite geographic and temporal differences, little is still known about the aetiology of NHL and the causal factors underlying the rise in incidence. A few risk factors have been established, including primary immunodeficiency disorders, HIV-infection, immunosuppressive medication following organ transplantation, certain autoimmune diseases and family history of NHL, but these factors only explain few new cases in the general population [3], [6]. Furthermore, improved cancer reporting, changes in NHL classification and increases in AIDS-associated lymphomas account for about half of the increased incidence [6]. Pesticides, hair dye, some industrial chemicals and diet have also been suggested as risk factors for NHL in some studies, while others do not find such associations [7], [8].

The rapid change in incidence rates argues for a substantial environmental influence, because genetic factors change at a much slower rate. If a risk factor is present in an area at a certain time period this would affect the people living in this area and potentially cause a higher than expected number of cases among these people. Space-time cluster analyses based on residential histories could therefore potentially reveal unknown environmental factors related to NHL, if such space-time clusters of NHL were to be found. A number of previous studies have used spatial analyses in an attempt to identify environmental risk factors associated with NHL, including investigations of spatial proximity of NHL cases to nuclear installations [9], to agricultural and industrial production [10], to a solid waste incinerator [11], and to a toxic industrial waste site [12]. Apparent associations were found in some of these geographic studies, but they have not lead to any plausible aetiological hypotheses about NHL. A few studies have examined spatial clustering of childhood NHL and leukaemia at more than one point in time e.g. the residential address at time of conception/birth and at time of diagnosis [13]–[15], providing inconclusive results. The inability of previous spatial studies to generate new hypotheses about the aetiology of NHL may partly be attributed to the failure of these studies to adequately account for human mobility. Latency periods between causative exposure and disease manifestation are likely for chronic diseases such as cancer, and therefore, it is crucial to take human mobility into account in the search for spatial clustering of cases related to environmental factors. A single study recently investigated space-time clustering of NHL in Detroit, Los Angeles, Seattle, and Iowa in the US and found that significant clustering mostly occurred 20 years before diagnosis [16]. The aim of the present study was to identify clustering of NHL in space and time in Denmark, using 33 years of residential histories. This exploratory space-time clustering analysis could lead to identification of environmental hazards. Given the heterogeneous malignancies that are captured by an NHL diagnosis, restricting the study population to those with origin in the lymph nodes or some of the major histological subtypes may produce more homogenous groups of cases [8].

## Materials and Methods

### Ethics Statement

The Danish Data Protection Agency (2007-41-0437) approved the study. In accordance with Danish law written consent was not obtained as the study was entirely register-based and did not involve biological samples from, or contact with study participants.

### Cases

NHL cases were identified in the population-based Danish Cancer Registry, to which it has been mandatory to report all new cancer diagnoses since 1987 [17]. We included all men and women diagnosed between 1999 and 2003 with diagnosis codes 200, 202 and 205 according to the 7^th^ Revision of the International Classification of Diseases. Only first, primary cancers were included, however previous diagnoses of non-melanoma skin cancer were allowed. The study included 3210 cases in total (1753 men and 1457 women). The choice of study period was mainly determined by data availability from the registers and our aspiration to include as much time as possible of the participants’ life-course. Sub groups of cases were classified according to the 3^rd^ Revision of International Classification of Diseases for Oncology. Topography code C77 was used to identify NHL cases with nodal origin, diffuse large B-cell lymphomas were defined by morphology code 9680.3 and follicular lymphomas were defined by morphology codes: 9690.3, 9691.3, 9695.3, and 9698.3.

### Controls

Controls were randomly selected from the Danish Civil Registration System [18] using density sampling and individually matched with cases by sex and date of birth (day, month and year). Further, controls were alive, living in Denmark and with no previous cancer diagnosis (except from non-melanoma skin cancer) at the date of diagnosis of the matched case. For the oldest cases (90 years of age or older), controls were matched by sex, birth month and year, only. The wider time range for matching in this age group was applied to ensure enough potential controls alive at the time of diagnosis. We selected two independent control groups with 3210 individuals in each group. The selection was carried out with replacement, which means that the controls have equal chance of being selected in the first control group as in the second. This study design allowed us to repeat all our analyses with a second group of controls and hereby verify our findings.

### Residential histories

We used the unique personal identification numbers of cases and controls to trace residential histories from 1971 to date of diagnosis of cases and their matched controls by record linkage with the Danish Civil Registration System. We identified 41,115 unique addresses, each with a unique identification number composed of a municipality code, a road code, and a house number. The date of moving in and leaving each residence was registered. The addresses were then linked to a register of all official addresses in Denmark, resulting in geographic coordinates for 40,410 (98%) of the residential addresses. In the geocoding procedure of the 40,410 addresses that matched with the register, 86% of the addresses of both cases and controls matched to the exact house. Additionally 4% matched to one of the neighbouring houses, and approximately 2% of the addresses matched to the centre of the road. The last 8% only matched at the municipality level, which means that the coordinates of the centroid of the municipality was assigned to these addresses. The administrative boundaries of the Danish municipalities were changed in 2007 and we used the municipalities that existed before 2007 in the present study. The average size of a municipality in Denmark before 2007 was 158 square kilometres. We did not exclude the least precise geocodes (municipality centroids) from the analyses, because they were uniformly distributed among cases and controls. Further, the ages as well as number of years prior to date of diagnosis of cases and their matched controls were calculated at the beginning and end of each residence. This enabled us to use different time scales in the spatio-temporal cluster analyses [19].

### Co-variates

A number of autoimmune diseases, HIV, AIDS and organ transplants increase the risk of NHL. In order to rule out potential confounding by these medical conditions, co-variates were derived for adjustment of the spatial analyses of NHL. The Danish National Patient Register holds information on all somatic hospital admissions in Denmark from 1977 and onwards, and since 1995, data on outpatient diagnosis and emergency patients have also been included. The 8^th^ Revision of the International Classification of Diseases was used to classify the diagnostic information in the register from 1977 to 1993, and the 10^th^ revision has been used since 1994 [20], [21]. Cases and controls were linked with the Danish National Patient Register by their personal identification number to obtain information on diagnoses and operations. These data were used to identify previous diagnoses of HIV, AIDS or AIDS-associated diagnosis, rheumatoid arthritis, celiac disease, systemic lupus erythematosus, Sjögren’s syndrome and organ transplants among cases and controls. We counted first diagnosis of autoimmune disease (rheumatoid arthritis, celiac disease, systemic lupus erythematosus, Sjögren’s syndrome) until one year before cancer diagnosis and diagnosis of HIV/AIDS or organ transplantation prior to date of cancer diagnosis whichever came first. The one year lag time for autoimmune diseases was applied to avoid possible inverse causality between these diseases and NHL (it should be the autoimmune disease that influences the development of NHL and not vice versa).

We combined autoimmune disease, HIV/AIDS and organ transplant into a single measure and used conditional logistic regression to obtain a risk estimate for the association with NHL. The risk estimate was converted into a probability, expressing the probability of being a case after a diagnosis of autoimmune disease, HIV/AIDS or organ transplantation. This probability was used for adjustment in the spatio-temporal analyses [22].

### Q-statistics

Jacquez et al. (2005) developed Q-statistics along with the software called SpaceStat for space-time cluster analysis of residential histories in case-control studies (BioMedware Inc., Ann Arbor, MI). Readers unfamiliar with the statistic may refer to the original work [23]. A short introduction to the method is given below.

Q-statistics is a novel approach that can take all locations over the entire life-course into account in the cluster analysis. Based on spatial neighbour relations this method evaluates local and global clustering at any moment in the residential histories of cases and controls. The spatial and temporal local case-control cluster statistic is given in equation 1:
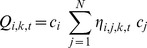
(1)


This quantity is the count, at time *t*, of the number of *k* nearest neighbours of case *i* that are cases, and not controls. *c_i_* and *c_j_* are case-control identifiers, which take the value1 if *i* is a case and 0 otherwise. The term 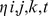 is a binary spatial proximity metric, which takes the value 1 when participant *j* is a *k* nearest neighbour at time *t* of participant *i*; and 0 otherwise. Since a given individual *i* may have *k* unique nearest neighbours, the *Q_ikt_* statistic is in the range 0..*k*. *Q_ikt_* is always 0 when *i* is a control. When *i* is a case, low values indicate cluster avoidance (e.g., a case surrounded by controls), and large values (near *k)* indicate a cluster of cases (e.g., a case surrounded by other cases). When *Q_ikt_*  =  *k*, at time *t* all of the *k* nearest neighbours of case *i* are cases.

This statistic is recalculated for each case every time there is a change in residence. *Q_ikt_* has a value for each residence at each and every time-geography of the residential histories. The value of *k* is specified by the user. The statistical significance of *Q_ikt_* is evaluated by use of randomizations. This is accomplished by holding the location histories for the cases and controls constant, and by then sprinkling the case-control identifiers at random over the residential histories, corresponding to the null hypothesis of no association between places of residence and case-control status. The randomization procedure is repeated over many iterations to build up the distributions of the Q-statistics under the null hypothesis. If information on covariates and other risk factors is available, the null hypothesis can account for them by employing the adjusted probabilities of being a case as calculated from logistic regression [22]. To calculate a p-value, the observed value of the Q-statistics for the observed data is then compared to the expected distribution obtained from the randomizations. The range of possible p-values is determined by the number of randomizations. In the present study we used 999 iterations generating a minimum p-value of 0.001.

Several different measures can be obtained from the Q-statistics. In the present study we used *Q_ikt_* and the duration-weighted version of *Q_ik_*, which means that residential addresses of longer time periods will be given greater weight than addresses where cases and controls only stay for shorter periods. *Q_ik_* is the sum of each individual’s *Q_ikt_* values and identifies which individuals tend to be centers of clusters over their life-course, but not when those clusters occur. *Q_ikt_* determines when and where an individual is a center of a local cluster.

Recently, our group conducted a simulation study to evaluate the performance of Q-statistics given the propensity for multiple testing and to explore the sensitivity of results to the choice of *k* nearest neighbours [24]. The study resulted in a guide for using and interpreting Q-statistics, which suggested that a cluster could be further evaluated as a possible true cluster if 4 or more significant cases were detected in the same area with a *Q_ik_* p = 0.001 and *Q_ikt_,* p≤0.05. We used this guide for the analyses performed in the present study. Analyses were carried out with *k*  =  15, since results from the simulation study, based on a Danish case-control dataset of similar size as the present study, indicated that a *k* of 15 performed well [24]. Further, we ran both unadjusted analyses and analyses adjusted for previous history of autoimmune disease, HIV/AIDS or organ transplantation. All analyses were conducted twice, first with one control group and then with the second control group. Three different time scales were applied: calendar year, age, and years prior to diagnosis. Finally, we analysed data with the two control groups combined in a 1:2 individually matched design. We also conducted separate analyses of different sub group of cases 1) NHL originating in the lymph nodes, 2) diffuse large B-cell lymphomas and 3) follicular lymphomas. In total, 54 runs of *Q* were conducted. In addition, we repeated selected analyses of potential clusters in SaTScan (version 9.1.1), to compare with results generated by an established cluster detection method [25], [26]. However, this method cannot account for human mobility, thus analyses were conducted on sub-sets of the original space-time data, which included only one location per individual. These time slices were selected to match statistically significant time periods identified by Q-statistics, the approach suggested in the simulation study of Sloan et al. (2012) [24]. We used a Bernoulli model in SaTScan, and the p-value for test of significance was obtained from Monte Carlo simulations (999 replications). We analysed elliptical clusters with a maximum cluster size of 10% of the total population.

## Results

The study included 3210 cases of NHL and 6420 controls divided into two separate control groups. NHL originated in the lymph nodes for 2339 (73%) of the cases. In addition, data included 1189 (37%) diffuse large B-cell lymphomas and 533 (17%) follicular lymphomas. The average age at diagnosis for all cases was 65 years, 64 years for cases with nodal origin, 66 years for diffuse large B-cell lymphoma and 59 years for follicular lymphoma. Both cases and controls lived at 4 different addresses, on average, in the period 1971–2003. A total of 122 (3.8%) cases and 74 (1.2%) controls had a history of HIV/AIDS, autoimmune disease or organ transplantation, providing a relative risk of NHL of 3.41 (95% confidence intervals 2.54–4.58), p-value < 0.0001, in association with a history of one of these conditions.

**Figure 1:**
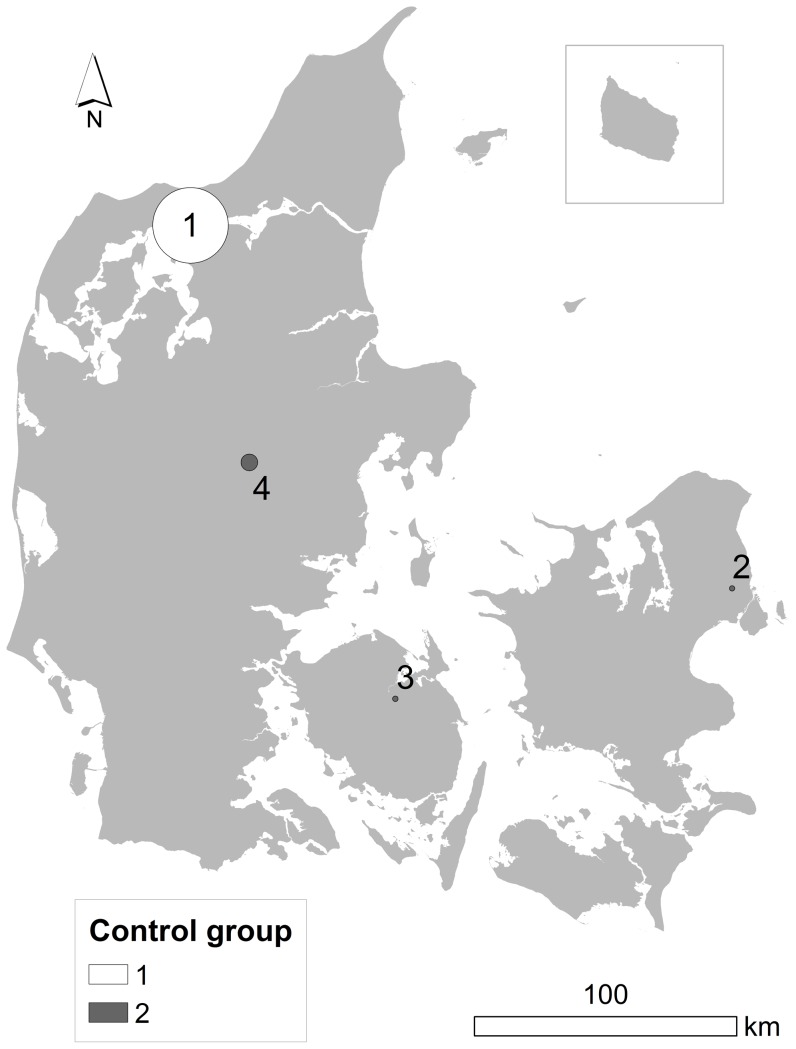
Map of space-time clusters of non-Hodgkin lymphoma in Denmark. Four areas showing statistically significant clustering of non-Hodgkin lymphoma cases in Denmark identified with Q-statistics, based on 15 nearest neighbours and unadjusted analyses. The circles indicate the extent of the clusters, not the number of cases comprising each cluster. None of these cluster regions were consistently found with both control groups.

When the first control group was applied, cluster area 1 was statistically significant with 6 cases, when participants were between 58 and 71 years old (table 1; figure 1). No other statistically significant clusters were identified, when control group one was used in the analyses.

**Table 1:** Results of unadjusted space-time cluster analyses performed in SpaceStat, based on 15 nearest neighbours, 999 permutations, and by three different time scales.

Study population	Time scale	Q_ik_ a	Q_ikt_^b^	Area, time^c^	Cluster area no. in figure 1 d
All cases, CG^e^ 1					
	Calendar year	1	–	–	–
	Age	6	6	Frøstrup, 58–71	1
	Years prior to diagnosis	2	–	–	–
All cases, CG 2					
	Calendar year	7	5	Copenhagen, Gladsaxe, 1974–1989	2
	Age	7	4	Odense, 42–48	3
	Years prior to diagnosis	8	4	Copenhagen, Gladsaxe, 27–13	2
All cases, CG 1 & 2					
	Calendar year	1	–	–	–
	Age	3	–	–	–
	Years prior to diagnosis	1	–	–	–
NHL (lymph nodes), CG 1					
	Calendar year	3	–	–	–
	Age	3	–	–	–
	Years prior to diagnosis	4	0	–	–
NHL (lymph nodes), CG 2					
	Calendar year	10	7	Silkeborg, 1974–1998	4
	Age	6	5	Silkeborg, 45–52	4
	Years prior to diagnosis	3	–	–	–
NHL (lymph nodes), CG 1 & 2					
	Calendar year	1	–	–	–
	Age	0	–	–	–
	Years prior to diagnosis	1	–	–	–

a The total number of statistically significant cases with a *Q_ik_* p-value of 0.001, which indicates the number of cases that are centers of clusters over their life-course. ^b^ Number of statistically significant *Q_ik_* (p  =  0.001) cases that also have significant *Q_ikt_* (p ≤ 0.05) and are members of a cluster of at least 4 cases. ^c^ Indicate where and when the cases tend to cluster.

d Refers to the map in figure 1, which shows the suggested clusters of NHL in Denmark based on the unadjusted analyses. ^e^ Control group.

Analyses with the second control group showed statistically significant clustering of 5 cases in 1974–1989 and 4 cases 27–13 years prior to diagnosis in cluster area 2 (table 1; figure 1). Cluster area number 3 (table 1; figure 1) had statistically significant clustering of 4 cases when participants were between 42 and 48 years old. Finally, statistically significant clustering of 7 cases of NHL originating in the lymph nodes was found in cluster area number 4 in 1974–1998 and of 5 cases when participants were between 45 and 52 years old (table 1; figure 1). For the histological subtypes, a single cluster of 4 diffuse large B-cell lymphomas was identified in the western part of Copenhagen in 1971–1995 and 1–16 years prior to diagnosis (results not shown). No other significant clusters were found with the second control group. None of the cluster areas were consistently identified for both control groups.

When we combined the control groups and repeated the analyses, no clustering of cases was observed.

**Figure 2:**
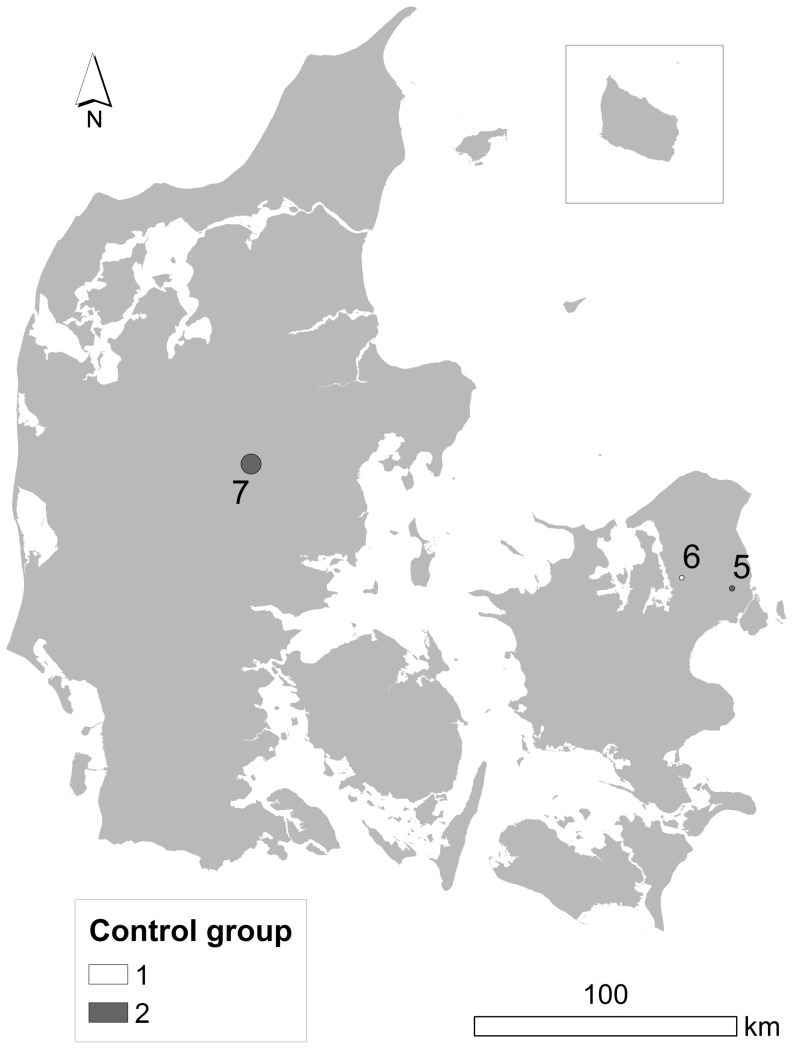
Map of space-time clusters of non-Hodgkin lymphoma in Denmark adjusted for potential confounding factors. Three areas showing statistically significant clustering of non-Hodgkin lymphoma cases in Denmark identified with Q-statistics, based on 15 nearest neighbours and adjusted analyses. The circles indicate the extent of the clusters, not the number of cases comprising each cluster. None of these cluster regions were consistently found with both control groups.

Results of the adjusted analyses are given in table 2 and shown in figure 2. When adjusting analyses for previous history of autoimmune disease, HIV/AIDS or organ transplantation cluster area number 1 and 3 (figure 1) from the unadjusted analyses disappeared. Area 5 and 7 (figure 2; corresponding to area 2 and 4 in figure 1) were identified in both the unadjusted and the adjusted analyses; however there were still no consistent findings across the two control groups. Area 6 (figure 2) was the only new area to emerge after the adjustment, with statistically significant clustering of 5 cases of NHL originating in the lymph nodes 18–2 years prior to diagnosis, but only when control group one was applied (table 2). As in the unadjusted analyses, combining the two control groups resulted in no clustering (table 2).

**Table 2:** Results of adjusted^a^ space-time cluster analyses performed in SpaceStat, based on 15 nearest neighbours, 999 permutations, and by three different time scales.

Study population	Time scale	Q_ik_ b	Q_ikt_^c^	Area, time^d^	Cluster area no. in figure 2 e
All cases, CG^f^ 1					
	Calendar year	0	–	–	–
	Age	1	–	–	–
	Years prior to diagnosis	2	–	–	–
All cases, CG 2					
	Calendar year	4	0	–	–
	Age	8	0	–	–
	Years prior to diagnosis	6	5	Copenhagen,Gladsaxe, 26–11	5
All cases, CG 1 & 2					
	Calendar year	1	–	–	–
	Age	2	–	–	–
	Years prior to diagnosis	1	–	–	–
NHL (lymph nodes), CG 1					
	Calendar year	2	–	–	–
	Age	2	–	–	–
	Years prior to diagnosis	7	5	Egedal, 18–2	6
NHL (lymph nodes), CG 2					
	Calendar year	5	0	–	–
	Age	6	4	Silkeborg, 43–52	7
	Years prior to diagnosis	8	5	Silkeborg, 24–3	7
NHL (lymph nodes), CG 1 & 2					
	Calendar year	0	–	–	–
	Age	0	–	–	–
	Years prior to diagnosis	1	–	–	–

a Adjusted for previous history of autoimmune disease, HIV/AIDS or organ transplantation.

b The total number of statistically significant cases with a *Q_ik_* p-value of 0.001, which indicates the number of cases that are centers of clusters over their life-course. ^c^ Number of statistically significant *Q_ik_* (p  =  0.001) cases that also have significant *Q_ikt_* (p ≤ 0.05) and are members of a cluster of at least 4 cases. ^d^ Indicate where and when the cases tend to cluster.

e Refers to the map in figure 2, which shows the suggested clusters of NHL in Denmark based on the adjusted analyses. ^f^ Control group.

**Figure 3:**
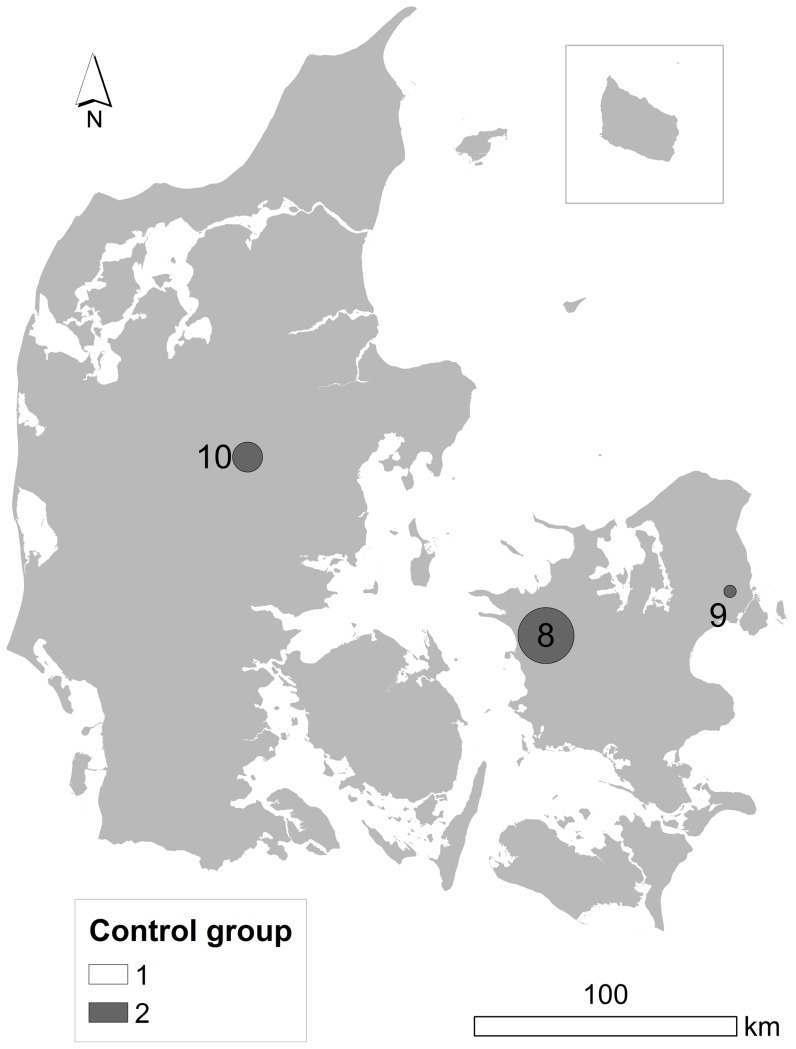
Map of space-only clusters of non-Hodgkin lymphoma in Denmark. One area showing statistically significant clustering of non-Hodgkin lymphoma cases (area no. 8), and two areas showing borderline clustering (area no. 9 and 10), based on spatial scan statistics of SaTScan and a maximum cluster size of 10% of the total population, using year when clusters were suggested by Q-statistics. None of these cluster regions were consistently found with both control groups.

Table 3 and figure 3 show results of analyses conducted with the spatial scan statistics of SaTScan. When this method was applied to the years where clusters were suggested by the Q-statistics, only a single cluster of 18 cases of NHL was statistically significant at age 45, using control group number two (table 3 and area 8 in figure 3). Further, using the second control group, a cluster of 24 NHL cases and one of 17 cases of NHL originating in the lymph nodes at age 62 and in 1981, respectively, were borderline significant (table 3, area 9 and 10 in figure 3). No clustering was observed when the first group of controls was used, thus results were not consistent across the two control groups.

**Table 3:** Results of analyses performed in SaTScan at selected time slices that match the time periods with the most significant space-time clusters found by Q-statistics.

Study population	Time scale	Time slice^a^	Lowest p-value^b^	N^c^	Area^d^	Cluster area no. in figure 3^e^
All cases, CG^f^ 1						
	Calendar year	1982	0.95	–	–	–
	Age	45	0.98	–	–	–
		62	0.25	–	–	–
	Years prior to diagnosis	15	0.82	–	–	–
All cases, CG 2						
	Calendar year	1982	0.28	–	–	–
	Age	45	0.04	18	Kalundborg	8
		62	0.08	24	Copenhagen/Gladsaxe	9
	Years prior to diagnosis	15	0.54	–	–	–
NHL (lymph nodes), CG 1						
	Calendar year	1981	0.64	–	–	–
	Age	48	0.93	–	–	–
NHL (lymph nodes), CG 2						
	Calendar year	1981	0.10	17	Silkeborg	10
	Age	48	0.12	–	–	–

a Indicates at which point in time we decided to apply the methods of SaTScan. ^b^ Indicates the most significant clusters detected by SaTScan. ^c^ Number of cases that comprise the most significant clusters detected by SaTScan. ^d^ Name of the area where SaTScan identified the cluster. ^e^ Refers to the areas shown on the map in figure 3. ^f^ Control group.

## Discussion

This large population-based case-control study found a number of statistically significant clusters of NHL in Denmark. However, none of these were consistent across the two control groups thus we interpreted the results as chance findings.

Denmark is a small country in northern Europe, with approximately 5.5 million inhabitants. It is characterised by both urban and rural areas, well-developed infrastructure and relatively homogenous population composition as regards race. The study was based on nation-wide registers and, therefore, included many different types of geographic areas e.g. rural and urban, high as low socioeconomic position, industrial and residential areas etc.

Apart from a single very recent study, the present study is the first examination of clustering of NHL in both space and time using residential histories. Cases were identified in the virtually complete high-quality population-based Danish Cancer Registry [17], [27], thus the study had very reliable case ascertainment. Furthermore, the Danish Civil Registration System provided an ideal frame for control selection and collection of residential addresses back to 1971[18]. The advantageous study design with two independent control groups was very helpful in the interpretation of the findings. We adjusted for HIV/AIDS, autoimmune disease or organ transplantation, because these conditions are associated with NHL and at least HIV/AIDS is likely to vary spatially and temporally; however the adjustment did not change our conclusions.

One limitation was that we did not have available information on participants’ work places; such data would have been useful, because clustering related to occupational exposures might have been detected. However, this is a shortcoming of almost all cluster studies. A larger limiting factor was related to computational time. Due to the large data sets of residential histories, a single analysis took up to 8 hours. Consequently we could not explore a wide range of different levels of *k* for the analyses, but had to rely on results from a simulation study when selecting *k* = 15 [24]. However, SaTScan spatial scan statistics, which search for clusters using variable-sized scanning windows, were used to confirm any potential cluster suggested by the Q-statistics.

The statistically significant cluster in Kalundborg (figure 3, area 8) identified by SaTScan had borderline significant Q*_it_* p-values in SpaceStat. Further, one borderline cluster detected by SaTScan confirmed the location of the Copenhagen-Gladsaxe cluster (figure 3, area 9), and another borderline SaTScan-cluster (figure 3, area10) agreed on both the time and location of the cluster found in Silkeborg by SpaceStat. Thus, results from analyses performed with the established spatial scan statistics of SaTScan to some extent confirmed the findings from the Q-statistic analyses done in the SpaceStat software. The fact that SaTScan neither found consistent clustering across the two control groups further strengthens our interpretation of the results as chance findings. The different results obtained with each of the two control groups indicate that our findings are due to random differences in the spatial and temporal distribution of controls in each of the control groups, as true clusters of cases would not depend on the control group applied. Chance findings due to random variation in the location of control group addresses would be minimized by increasing the number of controls. Indeed, when we combined the two control groups, all statistically significant clusters except one disappeared, which further indicates that the clusters identified by use of each of the control groups are driven by the distribution of the controls rather than the cases. Since the cases were identified and the controls were randomly selected from nation-wide registers, it is unlikely that selection bias influenced the results.

Additional numbers of controls would probably provide more consistent and reliable findings, which should be balanced against the increased computational time. This aspect could be evaluated in future studies.

The single previous space-time cluster study of NHL, based on residential addresses of 842 cases and 680 controls, found the strongest clustering when long lag times (20 years) were considered [16]. Our results do not confirm this since we found no consistent clustering of NHL when using either calendar year, age, or years prior to diagnosis as the underlying time scale and allowing for up to 33 years of lag time. Neither did we find consistent clustering among three different sub groups of cases.

The contradicting findings of the present study and that of Wheeler et al. (2011) could be due to several differences between the two studies. The present study had the advantage of complete enrolment (no selection bias) due to the use of the Danish Cancer Registry and Civil Registration System, whereas the former study had incomplete case-control participation. The present study also had nearly complete, objectively obtained information on residential addresses from the Civil Registration System and medical history from the National Patient Register. While the former study relied on self-reported interview data, which provided important additional information on potential risk factors for NHL, which the present study did not have. Although useful, these data could at the same time have introduced recall bias to the former study [16].

Further, the study by Wheeler et al. (2011) assessed residential history from birth, whereas the present study only had residential histories from 1971, where the average age of the study population was 36 years. Therefore, if exposures in early-life were important in the aetiology of NHL, it would not be revealed in the present study. The two studies also differed regarding statistical methods, covariates and case selection. The present study was restricted to first incident cases of NHL (with the exception of prior non-melanoma skin cancer), whereas the former study was not [16]. The reason for only including first primary cancer in the study was that secondary cancers could be a result of the treatment for the preceding cancer, thus allowing both first and second primary cancers would confuse the interpretation of the results. E.g. it would be difficult to determine if a detected cluster was related to potential environmental factors or to cancer treatment. Another factor that might complicate the interpretation of NHL cluster studies is familial aggregation of NHL, which could also cause space-time clustering of cases, as families tend to live in the same areas. Whether the underlying reason for familial clustering involves genetic or environmental factors would not be possible to decide based on the methods of the present study.

As we did not find consistent clusters of NHL in Denmark that could give clues to environmental factors, this could indicate that the rise in NHL incidence in Denmark is related to some general changes in life style or environment at the national level. Another possibility is that the cluster criteria used in the present study may be too conservative, thus underreporting clusters of NHL. On the other hand, SaTScan confirmed that there were no consistent clusters across the two control groups. Finally, we were not able to account for residential addresses before 1971 and have, therefore, not investigated clustering in early-life.

The present study utilized a novel method of cluster analysis in both space and time across residential histories and allowing adjustment for potential confounding factors. Accounting for human mobility in cluster analyses is especially important in studies of cancer because of long latency periods. Together with the high quality Danish registers and the “double-control group design” the new Q-statistics in SpaceStat provided ideal methods for a large-scale investigation of the spatial and temporal distribution of NHL in Denmark.

## Conclusions

We found no evidence for clustering of NHL in space and time using 33 years of residential histories, suggesting that if the rise in incidence of NHL in the second half of the 20^th^ century is a result of risk factors that vary across space and time, the spatio-temporal variation of such factors in Denmark is too small to be detected with the applied method. Our study also underlines the importance of a large control group in space-time cluster studies. We recommend that future geographic case-control studies employ study designs with two or more control groups in order to replicate within-study results.
